# Na_4_Fe^2+^Fe^3+^(PO_4_)_3_, a new synthetic NASICON-type phosphate

**DOI:** 10.1107/S1600536809009210

**Published:** 2009-03-19

**Authors:** Frédéric Hatert

**Affiliations:** a University of Liège, Laboratory of Mineralogy B.18, B-4000 Liège, Belgium

## Abstract

This paper reports the crystal structure of tetra­sodium diiron tris(phosphate), Na_4_Fe^2+^Fe^3+^(PO_4_)_3_, which has been synthesized hydro­thermally at 773 K and 0.1 GPa. The crystal structure has been refined in the space group *R* 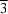 *c* and is identical to that of γ-NASICON. The heteropolyhedral framework is based on a regular alternation, in three dimensions, of corner-sharing PO_4_ tetra­hedra and FeO_6_ octa­hedra, constituting so-called ‘lantern units’ stacked along the *c* axis. The Na^+^ cations are distributed over two crystallographic sites: the six-coordinated Na1 site which lies between two ‘lantern units’, and the eight-coordinated Na2 site which lies at the same *z* value as the P site.

## Related literature

For general background, see: Hatert *et al.* (2006 ▶); Hatert & Fransolet (2006 ▶); Hatert (2007*a* ▶,*b* ▶). For related structures, see: Sljukic *et al.* (1969 ▶); Masquelier *et al.* (2000 ▶). For bond-valence calculations, see: Brown & Altermatt (1985 ▶). For the use of the pressure vessel, see: Tuttle (1949 ▶). For the standard used in the chemical analysis, see: Fransolet (1975 ▶). For software used to establish the space group, see: Le Page (1987 ▶).

## Experimental

### 

#### Crystal data

Na_4_Fe_2._(PO_4_)_3_
*M* *_r_* = 488.57
Trigonal, 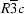

*a* = 8.9543 (9) Å
*c* = 21.280 (4) Å
*V* = 1477.6 (4) Å^3^
*Z* = 6
Mo *K*α radiation
μ = 3.68 mm^−1^
*T* = 293 K
0.08 × 0.05 × 0.03 mm

#### Data collection

Bruker *P*4 diffractometer
Absorption correction: ψ scan (*XSCANS*; Siemens, 1991 ▶) *T*_min_ = 0.693, *T*_max_ = 0.896
721 measured reflections
294 independent reflections
284 reflections with *I* > 2σ(*I*)
*R*_int_ = 0.021
3 standard reflections every 97 reflections intensity decay: 1.9%

#### Refinement

*R*[*F* ^2^ > 2σ(*F* ^2^)] = 0.052
*wR*(*F* ^2^) = 0.125
*S* = 1.40
294 reflections
35 parameters
Δρ_max_ = 1.70 e Å^−3^
Δρ_min_ = −0.97 e Å^−3^

### 

Data collection: *XSCANS* (Siemens, 1991 ▶); cell refinement: *XSCANS*; data reduction: *SHELXTL-Plus* (Sheldrick, 2008 ▶); program(s) used to solve structure: *SHELXS97* (Sheldrick, 2008 ▶); program(s) used to refine structure: *SHELXL97* (Sheldrick, 2008 ▶); molecular graphics: *ATOMS* (Dowty, 1993 ▶); software used to prepare material for publication: *SHELXTL-Plus*.

## Supplementary Material

Crystal structure: contains datablocks global, I. DOI: 10.1107/S1600536809009210/mg2067sup1.cif

Structure factors: contains datablocks I. DOI: 10.1107/S1600536809009210/mg2067Isup2.hkl

Additional supplementary materials:  crystallographic information; 3D view; checkCIF report

## Figures and Tables

**Fig. 1.**
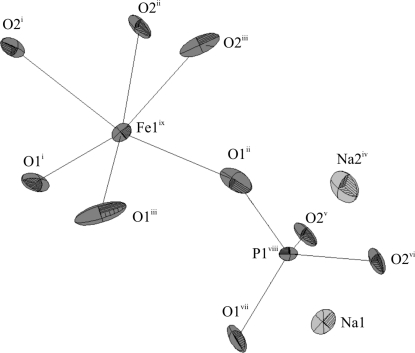
Fragment of the Na4Fe2+Fe3+(PO4)3 structure (50% displacement ellipsoids) showing the bonding environments of the Fe and P cations. [Symmetry codes: (i) 2/3 + x - y, 1/3 - y, 1/3 - z; (ii) 2/3 - x, 1/3 - x + y, 1/3 - z; (iii) 2/3 + y, 1/3 + x, 1/3 - z; (iv) x, -y - 1/3, z; (v) x, x - y, 1/6 -z; (vi) 1/3 + y, 2/3 - x + y, 1/6 - z; (vii) 2/3 - y, 1/3 + x - y, z - 1/6; (viii) -y, -x, z; (ix) x + 2/3, y + 1/3, 1/3 - z.]

**Fig. 2.**
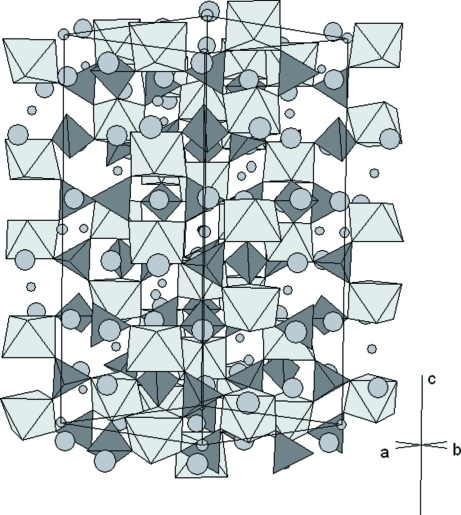
Polyhedral view of the Na4Fe2+Fe3+(PO4)3 structure. FeO6 octahedra are shown with light shading, PO4 tetrahedra with dark shading, Na1 as small circles, and Na2 as large circles.

## supplementary crystallographic information

### Comment

In the natural geological environment of granitic pegmatites, Na–Fe–Mn-bearing phosphates play important geochemical and petrological roles. The alluaudite group of minerals, with an idealized chemical composition Na_2_(Mn,Fe^2+^)_2_Fe^3+^(PO_4_)_3_, constitutes a good example of primary phosphates which can be used as geothermometer, or to constrain the oxygen fugacity which prevailed in granitic pegmatites (Hatert *et al.*, 2006). In order to better understand the crystallization conditions of iron-rich alluaudites in pegmatites, we decided to investigate the Na–Fe^2+^–Fe^3+^ (+PO_4_) ternary system by hydrothermal methods (Hatert & Fransolet, 2006). These experiments produced several new phosphates, which crystallized in the Na-rich part of the system and were investigated by single-crystal X-ray diffraction techniques (Hatert, 2007*a*,*b*). Starting from the composition Na_4_Fe^2+^Fe^3+^(PO_4_)_3_, the hydrothermal synthesis at 500°C and 0.1 GPa produced large pink crystals; their crystal structure is reported herein.

The crystal structure of the title compound has been refined in space group *R*3*c* and corresponds to that of centrosymmetric γ-NASICON-type phosphates (Sljukic *et al.*, 1969; Masquelier *et al.*, 2000). The heteropolyhedral framework is based on the regular alternation, in the three dimensions, of corner-sharing PO_4_ tetrahedra (P—O = 1.533–1.538 Å) and FeO_6_ octahedra (Fe—O = 2.010–2.130 Å; Fig. 1), and shows the stacking, along the *c* direction, of the so-called 'lantern units' (Masquelier *et al.*, 2000). The monovalent Na^+^ cations are distributed over two crystallographic sites: the 6-coordinated Na1 site (Na1—O = 2.402 Å) which lies between two 'lantern units', and the 8-coordinated Na2 site (Na2—O = 2.487–2.990 Å) which lies at the same *z* value as the P atom (Fig. 2).

Bond-valence sums were calculated for each ion using the parameters of Brown & Altermatt (1985). The P1 bond-valence sum is 4.99, and the O-atom bond-valence sums are within the normal acceptable range (1.93–2.10). The bond-valence sums also confirm that the Na1 and Na2 sites are filled by Na (0.98–1.19), and that the Fe1 site contains an equal amount of Fe^2+^ and Fe^3+^ (2.54).

A comparison with the crystal structure of γ-Na_3_Fe_2_(PO_4_)_3_ (Masquelier *et al.*, 2000) shows that the two phosphates are isostructural. However, the amount of Na in the title compound reaches 4 atoms per formula unit, and is higher than the Na-content of any other known NASICON-type phosphate. This high Na-content is necessary to maintain charge balance, since the Fe1 site is occupied by 50% Fe^2+^ and 50% Fe^3+^. Na atoms are located on the same positions as in γ-Na_3_Fe_2_(PO_4_)_3_, but the two Na1 and Na2 sites are completely filled by Na atoms in the title compound, whereas their occupancy factors are 0.85 (3) (Na1) and 0.72 (3) (Na2) in γ-Na_3_Fe_2_(PO_4_)_3_ (Masquelier *et al.*, 2000).

Masquelier *et al.* (2000) demonstrated that the size of the Na1 cavity is increased as this site is depopulated, and that this increase has a direct influence on the value of the *c* unit-cell parameter. This hypothesis is corroborated by our structural data, which show that a full occupancy of the Na1 site in Na_4_Fe^2+^Fe^3+^(PO_4_)_3_ induces small values for the Na1—O distances and for the *c* parameter (2.402 and 21.280 Å, respectively), while a partial occupancy of this site in γ-Na_3_Fe_2_(PO_4_)_3_ induces larger values for these parameters (2.500 and 21.808 Å, respectively; Masquelier *et al.*, 2000). The presence of significant amounts of Fe^2+^ in the Fe1 site of the title compound also induces a significant increase of the Fe1—O average bond length (2.070 Å), when compared to the Fe1—O bond length reported for γ-Na_3_Fe_2_(PO_4_)_3_ (2.002 Å; Masquelier *et al.*, 2000). This increase does not affect the *c* unit-cell parameter, but induces a significant increase of the *a* parameter, from 8.727 Å in γ-Na_3_Fe_2_(PO_4_)_3_ (Masquelier *et al.*, 2000), to 8.954 Å in the title compound.

### Experimental

The title compound was synthesized under hydrothermal conditions. The starting material was prepared by mixing NaH_2_PO_4_.H_2_O, Na_2_HPO_4_.2H_2_O, FeO and Fe_2_O_3_ in proportions 2:1:1:1/2. About 25 mg of the homogenized mixture was sealed into a gold tube with an outer diameter of 2 mm and a length of 25 mm, containing 2 mg of distilled water. The gold capsule was then inserted in a Tuttle-type pressure vessel (Tuttle, 1949) and maintained at a temperature of 500°C and a pressure of 0.1 GPa. After 3 d, the sample still in the gold tube in the autoclave, was quenched to room temperature in a stream of cold air. The synthesized phosphates consisted of large pink crystals of the title compound, associated with colourless crystals of maricite, NaFe^2+^(PO_4_), and with isometric black crystals of Na_7_Fe_4_(PO_4_)_6_.

A chemical analysis of the title compound has been performed with a CAMEBAX SX-50 electron microprobe (15 kV acceleration voltage, 20 nA beam current, analyst H.-J. Bernhardt, Ruhr-Universität Bochum, Germany). The standards used were graftonite from Kabira (sample KF16; Fransolet, 1975) (Fe, P), and jadeite (Na). The average of 6 point analyses gives P_2_O_5_ 42.47, FeO^*^ 16.94, Fe_2_O_3_^*^ 14.17, Na_2_O 24.47, total 98.05 wt. % (^*^ values calculated to maintain charge balance). The chemical composition, calculated on the basis of 3 P, corresponds to Na_3.96_Fe^2+^_1.18_Fe^3+^_0.89_(PO_4_)_3_.

### Refinement

A first refinement in space group *R*3 converged to a satisfactory model with *R*_1_ = 0.0328, but a check of these structural data with the program ADDSYM (Le Page, 1987) immediately allowed to identify supplementary symmetry elements, thus confirming the space group *R*3*c*. All atoms were refined anisotropically, and a preliminary refinement of the site occupancy factors of Na1 and Na2 confirmed that these sites were filled with Na atoms only. In the final refinement cycle, the Na1 and Na2 site occupancy factors were consequently constrained to 1.0.

### Crystal data

**Table:**

Na_3.96_Fe_2.07_(PO_4_)_3_	*D*_x_ = 3.294 Mg m^−^^3^
*M**_r_* = 488.57	Mo *K*α radiation, λ = 0.71073 Å
Trigonal, *R*3*c*	Cell parameters from 35 reflections
Hall symbol: -R 3 2"c	θ = 5.9–12.6°
*a* = 8.9543 (9) Å	µ = 3.68 mm^−^^1^
*c* = 21.280 (4) Å	*T* = 293 K
*V* = 1477.6 (4) Å^3^	Isometric crystal, pink
*Z* = 6	0.08 × 0.05 × 0.03 mm
*F*(000) = 1422	

### Data collection

**Table:**

Bruker P4 diffractometer	284 reflections with *I* > 2σ(*I*)
Radiation source: fine-focus sealed tube	*R*_int_ = 0.021
graphite	θ_max_ = 25.0°, θ_min_ = 3.3°
ω scans	*h* = −10→1
Absorption correction: ψ scan (*XSCANS*; Siemens, 1991)	*k* = −1→10
*T*_min_ = 0.693, *T*_max_ = 0.896	*l* = −25→1
721 measured reflections	3 standard reflections every 97 reflections
294 independent reflections	intensity decay: 1.9%

### Refinement

**Table:**

Refinement on *F*^2^	Primary atom site location: structure-invariant direct methods
Least-squares matrix: full	Secondary atom site location: difference Fourier map
*R*[*F*^2^ > 2σ(*F*^2^)] = 0.052	*w* = 1/[σ^2^(*F*_o_^2^) + (0.0415*P*)^2^ + 5*P*] where *P* = (*F*_o_^2^ + 2*F*_c_^2^)/3
*wR*(*F*^2^) = 0.125	(Δ/σ)_max_ < 0.001
*S* = 1.40	Δρ_max_ = 1.70 e Å^−^^3^
294 reflections	Δρ_min_ = −0.97 e Å^−^^3^
35 parameters	Extinction correction: *SHELXL97* (Sheldrick, 2008), Fc^*^=kFc[1+0.001xFc^2^λ^3^/sin(2θ)]^-1/4^
0 restraints	Extinction coefficient: 0.0027 (6)

### Special details

**Table:**

Geometry. All e.s.d.'s (except the e.s.d. in the dihedral angle between two l.s. planes) are estimated using the full covariance matrix. The cell e.s.d.'s are taken into account individually in the estimation of e.s.d.'s in distances, angles and torsion angles; correlations between e.s.d.'s in cell parameters are only used when they are defined by crystal symmetry. An approximate (isotropic) treatment of cell e.s.d.'s is used for estimating e.s.d.'s involving l.s. planes.
Refinement. Refinement of *F*^2^ against ALL reflections. The weighted *R*-factor *wR* and goodness of fit *S* are based on *F*^2^, conventional *R*-factors *R* are based on *F*, with *F* set to zero for negative *F*^2^. The threshold expression of *F*^2^ > σ(*F*^2^) is used only for calculating *R*-factors(gt) *etc*. and is not relevant to the choice of reflections for refinement. *R*-factors based on *F*^2^ are statistically about twice as large as those based on *F*, and *R*- factors based on ALL data will be even larger.

### Fractional atomic coordinates and isotropic or equivalent isotropic displacement parameters (Å^2^)

**Table:**

	*x*	*y*	*z*	*U*_iso_*/*U*_eq_	
Fe1	0.0000	0.0000	0.14926 (9)	0.0082 (8)	
P1	−0.3333	−0.3672 (3)	0.0833	0.0080 (9)	
Na1	0.0000	0.0000	0.0000	0.0129 (17)	
Na2	−0.3333	−0.0278 (7)	0.0833	0.0262 (13)	
O1	0.1950 (8)	0.2066 (9)	0.1913 (3)	0.0213 (15)	
O2	0.1869 (8)	0.0152 (7)	0.0838 (3)	0.0135 (14)	

### Atomic displacement parameters (Å^2^)

**Table:**

	*U*^11^	*U*^22^	*U*^33^	*U*^12^	*U*^13^	*U*^23^
Fe1	0.0070 (8)	0.0070 (8)	0.0105 (11)	0.0035 (4)	0.000	0.000
P1	0.0041 (15)	0.0109 (12)	0.0065 (14)	0.0021 (7)	0.0000 (10)	0.0000 (5)
Na1	0.009 (2)	0.009 (2)	0.020 (4)	0.0047 (12)	0.000	0.000
Na2	0.013 (3)	0.029 (2)	0.031 (3)	0.0065 (13)	−0.001 (2)	−0.0005 (11)
O1	0.015 (3)	0.032 (4)	0.013 (3)	0.008 (3)	−0.006 (2)	−0.008 (3)
O2	0.012 (3)	0.019 (3)	0.012 (3)	0.010 (3)	−0.004 (2)	−0.006 (2)

### Geometric parameters (Å, °)

**Table:**

Fe1—O1	2.010 (6)	Na1—O2^vii^	2.402 (5)
Fe1—O1^i^	2.010 (6)	Na1—O2^i^	2.402 (5)
Fe1—O1^ii^	2.010 (6)	Na1—O2^ii^	2.402 (5)
Fe1—O2^i^	2.130 (6)	Na1—O2^viii^	2.402 (5)
Fe1—O2	2.130 (6)	Na2—O2^i^	2.487 (8)
Fe1—O2^ii^	2.130 (6)	Na2—O2^v^	2.487 (8)
P1—O1^iii^	1.533 (6)	Na2—O2^ii^	2.493 (6)
P1—O1^iv^	1.533 (6)	Na2—O2^ix^	2.493 (6)
P1—O2^i^	1.538 (6)	Na2—O1^ix^	2.534 (6)
P1—O2^v^	1.538 (6)	Na2—O1^ii^	2.534 (6)
Na1—O2^vi^	2.402 (5)	Na2—O1^x^	2.990 (8)
Na1—O2	2.402 (5)	Na2—O1^xi^	2.990 (8)
			
O1—Fe1—O1^i^	101.7 (2)	O2—Na1—O2^viii^	109.0 (2)
O1—Fe1—O1^ii^	101.7 (2)	O2^vii^—Na1—O2^viii^	71.0 (2)
O1^i^—Fe1—O1^ii^	101.7 (2)	O2^i^—Na1—O2^viii^	180.00 (17)
O1—Fe1—O2^i^	165.5 (2)	O2^ii^—Na1—O2^viii^	109.0 (2)
O1^i^—Fe1—O2^i^	86.5 (2)	O2^i^—Na2—O2^v^	60.5 (3)
O1^ii^—Fe1—O2^i^	88.1 (2)	O2^i^—Na2—O2^ii^	68.1 (3)
O1—Fe1—O2	86.5 (2)	O2^v^—Na2—O2^ii^	128.6 (2)
O1^i^—Fe1—O2	88.1 (2)	O2^i^—Na2—O2^ix^	128.6 (2)
O1^ii^—Fe1—O2	165.5 (2)	O2^v^—Na2—O2^ix^	68.1 (3)
O2^i^—Fe1—O2	81.8 (2)	O2^ii^—Na2—O2^ix^	163.3 (4)
O1—Fe1—O2^ii^	88.1 (2)	O2^i^—Na2—O1^ix^	93.1 (2)
O1^i^—Fe1—O2^ii^	165.5 (2)	O2^v^—Na2—O1^ix^	70.0 (2)
O1^ii^—Fe1—O2^ii^	86.5 (2)	O2^ii^—Na2—O1^ix^	114.28 (19)
O2^i^—Fe1—O2^ii^	81.8 (2)	O2^ix^—Na2—O1^ix^	68.73 (19)
O2—Fe1—O2^ii^	81.8 (2)	O2^i^—Na2—O1^ii^	70.0 (2)
O1^iii^—P1—O1^iv^	109.6 (5)	O2^v^—Na2—O1^ii^	93.1 (2)
O1^iii^—P1—O2^i^	106.9 (3)	O2^ii^—Na2—O1^ii^	68.73 (19)
O1^iv^—P1—O2^i^	112.1 (3)	O2^ix^—Na2—O1^ii^	114.28 (19)
O1^iii^—P1—O2^v^	112.1 (3)	O1^ix^—Na2—O1^ii^	160.8 (4)
O1^iv^—P1—O2^v^	106.9 (3)	O2^i^—Na2—O1^x^	113.49 (19)
O2^i^—P1—O2^v^	109.2 (5)	O2^v^—Na2—O1^x^	154.86 (18)
O2^vi^—Na1—O2	180.0 (3)	O2^ii^—Na2—O1^x^	52.52 (18)
O2^vi^—Na1—O2^vii^	71.0 (2)	O2^ix^—Na2—O1^x^	112.9 (3)
O2—Na1—O2^vii^	109.0 (2)	O1^ix^—Na2—O1^x^	86.74 (13)
O2^vi^—Na1—O2^i^	109.0 (2)	O1^ii^—Na2—O1^x^	108.0 (2)
O2—Na1—O2^i^	71.0 (2)	O2^i^—Na2—O1^xi^	154.86 (18)
O2^vii^—Na1—O2^i^	109.0 (2)	O2^v^—Na2—O1^xi^	113.49 (19)
O2^vi^—Na1—O2^ii^	109.0 (2)	O2^ii^—Na2—O1^xi^	112.9 (3)
O2—Na1—O2^ii^	71.0 (2)	O2^ix^—Na2—O1^xi^	52.52 (18)
O2^vii^—Na1—O2^ii^	180.0 (3)	O1^ix^—Na2—O1^xi^	108.0 (2)
O2^i^—Na1—O2^ii^	71.0 (2)	O1^ii^—Na2—O1^xi^	86.74 (13)
O2^vi^—Na1—O2^viii^	71.0 (2)	O1^x^—Na2—O1^xi^	82.0 (3)

Symmetry codes: (i) −*x*+*y*, −*x*, *z*; (ii) −*y*, *x*−*y*, *z*; (iii) −*x*+*y*−1/3, *y*−2/3, *z*−1/6; (iv) *x*−*y*−1/3, *x*−2/3, −*z*+1/3; (v) *x*−*y*−2/3, −*y*−1/3, −*z*+1/6; (vi) −*x*, −*y*, −*z*; (vii) *y*, −*x*+*y*, −*z*; (viii) *x*−*y*, *x*, −*z*; (ix) *y*−2/3, *x*−1/3, −*z*+1/6; (x) *x*−1/3, *x*−*y*+1/3, *z*−1/6; (xi) −*x*−1/3, −*y*+1/3, −*z*+1/3.
